# Determinants of Selected Cardiovascular Diseases among Adult Patients at Cardiac Clinic of Debre Berhan Referral Hospital, Ethiopia: Unmatched Case-Control Study

**DOI:** 10.1155/2020/7036151

**Published:** 2020-05-19

**Authors:** Shenkut Aragaw, Esubalew Tesfahun, Behailu Tariku Derseh, Betegiorgis Mamo

**Affiliations:** ^1^Public Health Department, College of Health Sciences, Debre Berhan University, Debre Berhan, Ethiopia; ^2^Department of English, College of Social Sciences, Debre Berhan University, Debre Berhan, Ethiopia

## Abstract

**Background:**

Africans are experiencing a rapid epidemiological transition characterized by urbanization and lifestyle changes, which are thought to contribute to increased incidence and prevalence of cardiovascular diseases (CVDs) in many African countries, including Ethiopia. Despite this, however, there is scarcity of evidence on cardiovascular disease risk factors among adults in the current research setting. This study thus aimed at assessing determinants of selected cardiovascular diseases among adult patients at Debre Berhan Referral Hospital (DBRH). *Methodology*. An unmatched case-control study was conducted on 143 newly diagnosed patients with CVDs and 286 controls at the cardiac clinic of DBRH from June to September 2017. Primary data were collected using the WHO-STEPS wise structured questionnaires. Multiple logistic regression analysis was used to identify potential risk factors for cardiovascular diseases at *p* values < 0.05.

**Result:**

The mean age of study participants is estimated as 45.5 ± 13.8 and ranges from 25 to 64 years. Sixty-one (42.7%) of cases and 147 (51.4%) of controls are males. Half of the cases (49.9%) had ischemic heart diseases (IHD), and 44.1% of cases had hypertensive heart disease (HHD), whereas the rest had chronic valvular heart disease (CRVHD) (4.2%) and peripheral and vascular disease (2.1%). This study identified older age as a risk factor for CVD: age group 35–44 years (adjusted odds ratio (AOR) = 2.20; 95% CI: 1.05–4.62), 45-54 years (AOR = 4.23; 95% CI: 2.19–8.16), and 55-64 years (AOR = 5.98; 95% CI: 3.26–10.98). Other risk factors were smoking history (AOR = 9.52; 95% CI: 2.12–42.8), low level of physical activity (AOR = 2.19; 95% CI: 1.10–5.02), and higher waist circumference (AOR = 2.75; 95% CI: 1.16–6.56).

**Conclusion:**

This study has demonstrated that the most frequent risk factors for CVD were older age, cigarette smoking, physical inactivity, and abdominal obesity. Therefore, behavior change communication focusing on lifestyle modification including regular physical activities, smoking cessation, and a balanced diet should be strengthened.

## 1. Introduction

Cardiovascular diseases (CVDs) as a part of noncommunicable diseases (NCDs) are a group of disorders of the heart and blood vessels and includes (not limited to) coronary heart disease (CHD), cerebrovascular disease, raised blood pressure, peripheral artery disease, rheumatic heart disease, congenital heart disease, and heart failure. These components of CVDs lead to heart attack and stroke, usually an acute event, and they are mainly caused by a blockage that prevents blood flowing from the heart to the brain. The most common reason for this obstruction is the building up of fat deposit in the inner wall of blood vessels that supply to the heart or brain [1]. Currently, research evidence showed that NCDs are increasing globally and threatens human lives. Specifically, in the 21^st^ century, because of globalization, there has been a paradigm shift in people's lifestyle and modernization across different regions and subregions in the globe, and such paradigm shift has contributed to the rapid increase of CVDs worldwide [2].

CVDs are one of the major problems among NCD killing people around the world [3]. According to the World Health Organization (WHO) report of 2015, CVDs were the leading cause of deaths globally, and it killed 17.5 million people in 2012, which represented 31% of all global deaths [4, 5]. Moreover, the risk factors for NCDs are unacceptably increasing in low- and middle-income countries including Africa [1, 6, 7]. The Heart Federation on the global strategy and diet, physical activity, and health showed that in 2015 people with CVDs had behavioral risk factors which include unhealthy diet, physical inactivity, tobacco use, and harmful alcohol consumption. These risk factors had an effect on increase in blood glucose, blood pressure, blood lipids, and overweight and obesity [6–8].

In low- and middle-income countries (LMICs), the magnitude of chronic diseases including CVD is increasing and creating a public health catastrophe [9]. At the same time, the threat of communicable and poverty-related diseases (malaria, infant mortality, cholera, and malnutrition) still exists in developing countries, including Africa [10–13]. For instance, in South Africa, CVD is the second leading cause of death after HIV/AIDS accounting for up to 40% of deaths among adults [14]. This double burden of communicable and NCDs has long-term public health impacts as it undermines healthcare systems [10].

Sub-Saharan Africa (SSA), consisting of those countries that are fully or partially located south of the Sahara Desert, is currently experiencing one of the most rapid epidemiological transitions characterized by increasing urbanization and changing lifestyle factors [10], which in turn have raised the incidence of NCDs, especially CVD [15]. Studies indicated that urbanization and economic development have also led to the emergence of a nutritional transition marked by a shift to a higher caloric content diet and/or reduction of physical activity [16]. The epidemiological and nutritional transitions together create enormous public health challenges, and failure to address such health problems may impose a significant burden on the health sector and the economy of SSA countries [10]. SSA countries, similar to most developing countries, often do not have the public health infrastructure and finances necessary to address both communicable and poverty-related illnesses and chronic illnesses [10]. In addition, there is reluctance on the part of health-funding agencies and policymakers to divert scarce resources away from communicable diseases into other areas of disease burden, such as NCDs [12, 17]. However, in SSA, NCDs such as CVD are anticipated to eclipse communicable and poverty-related diseases as the leading cause of mortality and disability [5, 18]. Research evidence also suggests that the increasing burden of chronic diseases has grave consequences for public health service because very few people will seek treatment and, as such, has led to high morbidity and mortality rates from potentially preventable diseases [19]. In this regard, a lack of resources in healthcare settings has serious consequences for the quality of disease management interventions for people with chronic illnesses. In addition to the increase in the burden of CVDs in low- and middle-income countries, there is scarcity of data that shows risk factors for CVDs [20].

In Ethiopia, healthcare resources are currently overwhelmed by public health threats, including HIV/AIDS, tuberculosis, and malaria. At the same time, the rapid urbanization of this country has created a change in people's lifestyles in terms of nutrition, physical activities, and behaviors such as smoking, alcohol, and drug use among urban dwellers, which may impact the risk of developing CVD. It is thus time to address NCDs including CVDs among adults [21].

A study done at Jimma University Specialized Hospital (JUSH) showed that Rheumatoid Heart Disease was the leading cause of heart diseases, which comprised 32% of patients followed by hypertensive heart disease (HHD, 24%) and cardiomyopathy that showed the shifting of epidemiology of heart diseases. Such shifting was double burden of diseases [22, 23]. It is also reported in the study that hospital beds in Ethiopia were mostly occupied with CVD and related cases [24, 25].

In view of the findings of the above-mentioned study, the present study aimed at filling in the existing research gap by examining the assumptions that observed risk factors in the research setting, such as physical activity, poor nutrition, risky alcohol behavior, cigarette smoking, obesity, diabetes mellitus, and high cholesterol which may contribute to the predictor of CVDs to the extent that needs public health attention.

## 2. Methodology

### 2.1. Study Design and Setting

A facility-based unmatched case-control study was conducted on patients at the cardiac follow-up clinic of Debre Berhan Referral Hospital from June to September 2017.

### 2.2. Sample Size and Sampling Procedure

For this unmatched case-control study, the sample size was determined by assuming the ratio of cases and controls (1 : 2); after conducting a pilot study on the percent of control exposed in the study setting considering alcohol consumption as a factor, we got 18% based on the following assumptions: power as 80% (*Z*_*β*_ = typically = 0.80); 5% of significance level (*Z*_*α*/2_ = 1.96); the ratio of control to case = 2; and the proportion exposed in the control group in the study setting was 18% (based on pilot study conducted, taking alcohol as exposure variable). To get the proportion of cases exposed, we use the following: *P* case exposed = OR∗*P* of control exposed; *P* of control exposed = *P* cases exposed∗2 = 0.18∗2.0 = 0.32; and average proportion exposed = [(0.32 + 0.18)/2] = 0.25. We use the above element in the following formula:

(1) $$\begin{array}{c} n=\left(\frac{r+1}{r}\right)\frac{\bar{P}\left(1-\bar{p}\right){\left(Z_{\beta }+Z_{\alpha /2}\right)}^{2}}{{\left(P1-P2\right)}^{2}}, \end{array}$$

where, *P*1 is proportion of control, *P*2 is proportion of cases, and *r* is ratio of control to cases; *n* = 130. Therefore, a total of 130 cases and 260 controls were estimated. Adding a 10% nonresponse rate, a sample of 429, (143) cases and (286) controls were selected [26].

#### 2.2.1. Selection of Cases

Cases were adult patients who had a confirmed diagnosis of CVDs attending a regular chronic disease follow-up clinic in Debre Berhan Referral Hospital with a first diagnosis starting since June 2016. CVDs are conditions that affect the structures or function of our heart. In this study, we identified ischemic heart diseases (IHD), hypertensive heart disease (HHD), chronic valvular heart disease (CRVHD), and peripheral and vascular disease. These diseases were diagnosed based on the standard hospital procedures: (I) two or more electrocardiogram (ECG) showing specific changes; (II) an ECG showing probable changes plus abnormal cardiac injury enzymes; or (III) typical symptoms such as a retrosternal pain plus abnormal enzymes [26]. Eligible cases were all adult patients who were on chronic illness follow-up clinic registered since June 2016 and available at the time of data collection. Consecutive sampling technique was used to include cases.

#### 2.2.2. Selection of Controls

A control is defined as an individual who visited the hospital for other than cardiovascular diseases. Simple random sampling technique was used to recruit controls from outpatients who were attending the hospital for correction of refractive errors, routine Pap smear, elective minor surgery, hemorrhoids, hernia surgery, and minor dermatological disorder. For the selection of control, a person's prior history regarding CVD was asked, and it was assured that they had never been admitted to the hospital or taken treatment for CVDs [26]. Moreover, we excluded outpatients with hypertension and diabetes mellitus since risk factors for these diseases and the cases might be similar.

### 2.3. Data Collection Techniques

The data collection was carried out among the alternative instrument tools for CVD risk factors based on the WHO-STEPS wise approach for the study of risk factors for chronic diseases (STEPS), which was developed by the World Health Organization. It was intended to serve as an entry point for low- and middle-income countries for the study of chronic diseases and their risk factors. STEPS were the sequential process involving the collection of data on selected risk factor with the questionnaire and basic physical measurements [5]. The first step was questionnaire-based assessment: This approach helps to gather information about variables like socioeconomic status, tobacco and alcohol use, measures of nutritional status, and physical inactivity. This step serves two purposes: consideration of the local context and comprehensiveness of the questionnaire to address the set objectives. The second step was simple physical measurements: It is a follow-up to the first step with the inclusion of simple physical measurements, such as height, weight, waist circumference, and blood pressure. Optional or new questions are added to the instrument because they are deemed locally important. All the modifications are done in accordance with the STEPS with participants standing without shoes and wearing light clothing. Weight measuring scales were checked and adjusted at zero levels between each measurement. Weight was recorded to the nearest 100 grams. Heights of subjects were measured to the nearest 0.5 centimeter, using a standard meter with subjects standing in the upright position and without shoes on their feet. To ensure that an upright position was maintained, each participant was told to stand straight forward, while the data collector positioned the head, so that the temporomandibular joint is at level with the eye and both heels on the ground, before taking the height measurement. Participant blood pressure was measured twice in a sitting position using a standard mercury sphygmomanometer BP cuff with the appropriate cuff size that covers two-thirds of the upper arm after the participant rest for at least five minutes and no smoking or caffeine 30 minutes before measurement. The second measurement will take five to ten minutes after the first measurement if the first measurement was inclination to hypertension. Finally, the average of two BP measurements was used to determine the status of the participant's blood pressure.

Body mass index (BMI) was calculated as weight in kilograms over height in meters squared (weight (kg)/(height (m))^2^). Waist circumference was measured at the level of the iliac crest using a nonelastic tape measure. Hip circumference was measured at the maximum circumference of the hip. The waist-to-hip ratio (WHR) was calculated as a ratio of waist and hip circumference. The global physical activity questionnaire section of the STEPS instrument was used for the assessment of physical activity. The instrument looks into three major domains; these were day-to-day activities: work (including domestic work), transport, and recreational activities. The level of total physical activity was subsequently classified into high, moderate, or low using the analysis guideline provided along with the STEPS instrument [27].

### 2.4. Data Quality Assurance

The questionnaire which was originally developed in English was translated to Amharic language and back-translated to English to ensure its consistency. Before data collection, the instrument was pretested by taking 5% study subjects for the feasibility of the questionnaires. Data recording quality was checked by the end of each day of data collection, for consistency, completeness, clarity, and accuracy; also during data processing, the information was checked for completeness and internal consistency. Data was collected by four clinical nurses. A one-day training and practical demonstration on interview techniques and measurement procedures was given to data collectors by supervisors.

### 2.5. Data Processing and Analysis

The data coding was done at the end of each day of data collection and recorded later where necessary. Data was entered into the computer using the Epi Info™ (© 7.2.0.1, CDC, 2016) statistical package, which was also used to clean the data. Frequencies and a random independent check were used to check for accuracy of data entry. Frequency distribution, percentage, tables, and charts were used to show the results of univariate analysis. Cross-tabulations, chi-square tests, *p* values, odds ratios, and 95% confidence intervals were used to present results of bivariate analysis. Factors with *p* < 0.25 in the bivariate analysis were selected and used in the multiple logistic regression analysis to adjust the confounders [28]. To identify the independent predictors of cardiovascular diseases, multivariate analysis was conducted via logistic regression analysis which also helps to control for potential confounders in the analysis. Statistical significance was declared at a *p* < 0.05.

## 3. Result

### 3.1. Sociodemographic Characteristics of Study Participants

A total of 143 cases and 286 controls were included in the study. Among the cases, 61 (42.7%) were male and 82 (57.3%) were female. The mean age of the case was 51.43 (SD ±12.076), regarding religious affiliation, and 141 (98.6%) of cases and 267 (93.4%) of controls were orthodox. Most cases, 91 (63.6%), and controls, 214 (74.8%), were married. The educational status of most cases 87 (60.8%) and controls 113 (39.5%) was illiterate. Regarding income, more than half 79 (55.2%) of cases and 115 (40.2%) of controls were included having a monthly income of less than 500 ETB. Concerning occupational status, 60 (42%) of cases and 117 (40.9%) of controls were farmers.

### 3.2. Chi-Square Test of Independence for Selected Covariates

A chi-square test of independence was performed to examine the relationship between smoking and the probability to develop CVDs. The relation between these variables was significant, *χ*^2^ (1, *N* = 429) = 6.17, *p* = 0.003. Smokers were more likely to develop cardiovascular diseases than nonsmokers. Likewise, the level of physical activity was statistically significant with cardiovascular diseases, *χ*^2^ (2, *N* = 429) = 9.64, *p* = 0.008. Study participants with low intensity of physical activity were more likely to develop CVDs than participants whose physical activity intensity is high (Table 1).

### 3.3. Determinants of Cardiovascular Diseases

Multivariable logistic regression showed that smokers are 9.5 (AOR = 9.52; 95% CI: 2.12, 42.8) times more likely to develop CVDs than nonsmokers. Similarly, respondents who perform low intensity of physical activity are 2.19 (AOR = 2.19; 95% CI: 1.10, 5.02) times at higher risk of having CVD compared to study participants engaged at a high level of physical activity (Table 2).

## 4. Discussion

This section summarizes the findings for the risk factors of CVD among patients in the cardiac clinic of DBRH which includes distribution of risk factors of CVD such as behavioral and biological factors among CVD cases and examining determinant of CVD among adults.

This study revealed that age is the most important risk factor for the development of cardiovascular diseases. Several studies conducted in Ethiopian, and 13 SSA countries and India showed that there is strong relationship between age and cardiovascular diseases [7, 21, 27, 29]. However, this study is inconsistent with the finding from India with the same study design [26]. The difference may be due to the aging of the population which makes people vulnerable to chronic diseases at older age in our study setting and increased vulnerability due to lifestyle changes.

Smoking increases the risk of atherosclerosis, platelet aggregation, and vascular osculation [30]. Accordingly, in our study, being a smoker is one of the strongest predictors of CVDs. This was in line with a study done in India [26, 31] which reported the association of smoking with the component of CVD, which was coronary artery disease compared to a nonsmoker [32].

Regarding physical activity level, patients whose physical activity intensity is low (<600 MET-minute per week) were two times more likely to develop CVD than those who had a high level of physical activity (>3000 MET-minute per week). This finding is consistent with studies conducted by WHO (2011) in the Horn of Africa including Ethiopia, which stated that daily physical activity minimizes the emerging and development of CVDs and its sequence through its effect on body weight, insulin sensitivity, and blood pressure control [26, 32–34].

There was no significant association between alcohol consumption and CVDs in this study. This is inconsistent with reports from previous studies in different parts of the world, such as the one conducted by Ram [26]. Similarly, study done by Jamila in Nigeria [35]. However, the proportion of alcohol consumption in our study among cases (85.8%) and controls (85.3%) was high compared to the national strategic action plan of Ethiopia (2014–2016), 45% of women and 53% men reported drinking alcohol at some point in their life [33]. The range of proportion of alcohol consumption reflects the different sociocultural norms of parts of the country.

Waist circumference (WC) indicates abdominal obesity and has been shown to be a risk factor for CVDs [7, 26]. In our study among biological risk factors, high waist circumference was found to be positively associated with CVDs. Those who had higher WC are more likely to develop CVDs than respondents who had normal WC. This finding is consistent with a previous study conducted in Tigray and found that waist circumference is statistically associated with cardiovascular diseases [32].

In summary, for CVDs, modifiable and easily measurable risk factors could be reliably used to predict the future burden of the diseases and to measure the effectiveness of public health interventions [33]. This suggests that the increased risks of cardiovascular diseases among chronically ill patients in our study setting may be preventable through lifestyle interventions along with careful use of medicines. Thus, smoking, obesity, and physical inactivity as predictors of CVDs are modifiable and preventable. Moreover, health promotion activities at health facility level should be strengthened to reduce the significant proportion of premature morbidity and mortality due to CVDs.

This case-control study, even if it identified the common predictors of CVDs, it has a number of limitations. Since it is a facility-based study, the cases and controls may not be the representative of the catchment population. As the study was carried out with a postgraduate public health student, we purposefully included interviewing and simple physical measurements, but we did not use biochemical measurements due to scarcity of reagents and time.

## 5. Conclusion

The burden of risk factors for CVDs is increasing in developing countries including Ethiopia. In this study, being an older age, smoking cigarette, being physically inactive, and being centrally obese were significantly associated with CVDs. Simple and client-friendly intervention programs aimed at preventing and modifying the risk factors in persons prone to CVDs should be incorporated into the healthcare system of Ethiopia to recognize them early and effectively manage them. Moreover, behavior change communication focusing on modifiable predictors of CVDs should be preached for the general community. This study highlighted the need for further study to determine the burden of the risk factors of CVDs at community setting.

## Figures and Tables

**Table 1:** Distribution of behavioral and physical measurement among study participants in Debre Berhan Referral Hospital, North Shoa, Ethiopia, 2017.

Characteristics	Cases	Controls	*χ* ^2^ value	*p* value
Past smokers				
Yes	08	04	6.17	0.013^∗^
No	135	282		
Ever used alcohol				
Yes	60	220	51.41	<0.001^∗^
No	83	66		
Waist circumference				
Normal	137	284	4.6	0.03^∗^
Above normal	06	02		
Body mass index				
≥30 kg/m^2^	13	15	2.37	0.31
25–30 kg/m^2^	15	29		
<25 kg/m^2^	115	242		
Weight-to-hip ratio				
Normal	11	6	6.44	0.01^∗^
Above normal	132	280		
Level of physical activity				
High (>3000 MET)	43	85		
Moderate (600-3000 MET)	76	180	9.64	0.008^∗^
Low (<600 MET)	24	21		
Number of fruit serving per week				
<5 servings	129	254	0.19	0.65
≥5 servings	14	32		
Number of veg servings per week				
<5 servings	141	279	0.127	0.72
≥5 servings	02	07		

BMI: body mass index; WHR: weight-to-height ratio.

**Table 2:** Multivariate logistic regression analysis of potential risk factor for CVD in Debre Berhan Referral Hospital, North Shoa, 2017.

Characteristics	Cases (*n*)	Controls (*n*)	COR, 95% CI	*p* value	AOR, 95% CI
Sex					
Male	61	147	1.42 (0.95, 2.13)^∗^	0.088	—
Female	82	139	1		
Age group					
<34	17	106	1		1
35-44	18	5	0.45 (0.22, 0.96)^∗^	0.037	2.20 (1.05, 4.62)^∗∗^
45-54	38	56	0.24 (0.22, 0.45)^∗^	<0.001	4.23 (2.19, 8.16)^∗∗^
>54	70	73	0.16 (0.09, 0.31)^∗^	<0.001	5.98 (3.26, 10.98)^∗∗^
Marital status					
Single	23	55	1		
Widowed	29	17	1.02 (0.75, 5.90)	0.952	—
Married	91	214	4.08 (1.89-8.82)^∗^	<0.001	
Average income					
<Q1	79	115	1		1
Q1-Q2	36	62	1.18 (0.72, 1.95)	0.510	1.28 (0.69, 2.22)
Q2-Q3	17	52	2.10 (1.13, 3.89)^∗^	0.019	1.61 (0.76, 4.17)
≥Q3	11	57	3.56 (1.76, 7.21)^∗^	<0.001	3.22 (1.35, 7.69)^∗∗^
Ever smokers					
Yes	8	4	4.17 (1.24, 4.12)^∗^	0.021	9.52 (2.12, 42.8)^∗∗^
No	135	282	1		1
Physical activity					
Low	24	21	2.23 (1.13, 4.51)^∗^	0.021	2.19 (1.10, 5.02)^∗∗^
Moderate	76	180	0.84 (0.53, 1.32)	0.435	0.84 (0.49, 1.43)
High	43	85	1		1
Waist circumference					
Raised	17	19	1.89 (1.24, 31.21)^∗^	0.045	2.75 (1.16, 6.56)^∗∗^
Normal	126	267	1		1
Weight-to-hip ratio					
Raised	11	6	1.19 (0.65, 2.19)^∗^		—
Normal	132	280	1	0.01	

WC: waist circumference; BMI: body mass index; WHR: waist-hip ratio; CI: confidence interval. ^∗^Statistically significant. ETB: Ethiopian birr; WC: normal in women <88 cm, normal in men <102 cm; WHR: greater than 1.0 in men or greater than 0.85 in women.

## Data Availability

The type of data used to support the findings of this study are included within the article.

## Abbreviations

CRVHD: Chronic valvular heart disease
CVD: Cardiovascular disease
CHF: Congestive heart failure
DALY: Disability-adjusted life year
DBRH: Debre Berhan Referral Hospital
DBP: Diastolic blood pressure
DSS: Demographic surveillance systems
GBD: Global burden of diseases
HHD: Hypertensive heart disease
HIV: Human immune viruses
IHD: Ischemic heart diseases
MET: Metabolic equivalent
OR: Odds ratio
RHD: Rheumatic heart disease
SPSS: Statistical Package for Social Science
WHO: World Health Organization
WHR: Weight-to-height ratio
WC: Waist circumference.
